# Impact of COVID-19 on sputum isolates and hospital outcomes among patients with pneumonia in Sheffield, United Kingdom: a retrospective cohort study

**DOI:** 10.3389/fpubh.2026.1816789

**Published:** 2026-06-30

**Authors:** Patrick Copley, Emmanuel Firima, Natasha James, Matthew Saunders, David Partridge, Joby Cole

**Affiliations:** 1 Department of Infection and Tropical Medicine, Royal Hallamshire Hospital, Sheffield Teaching Hospitals National Health Service Foundation Trust, Sheffield, United Kingdom; 2 School of Medicine and Population Health, The University of Sheffield, Sheffield, United Kingdom; 3 The Florey Institute, University of Sheffield, Sheffield, United Kingdom; 4 Department of Clinical Microbiology, Sheffield Teaching Hospitals NHS FT, Sheffield, United Kingdom

**Keywords:** community acquired pneumonia, COVID, epidemiology, pneumonia - clinical features and management, *Streptococcus pneumonia*

## Abstract

**Background and aim:**

The COVID-19 pandemic caused by SARS-CoV-2 severely affected healthcare globally. Strong links have been suggested between the pandemic and changes in the epidemiology of organisms responsible for non-COVID-19 pneumonia. In this study, we examined temporal changes in organisms recovered from sputum culture, integrating laboratory data from all submitted sputum specimens with clinical data from a subset of patients diagnosed with community-acquired pneumonia (CAP).

**Method:**

A retrospective study was conducted from August 2017 to May 2024 using data derived from samples from healthcare centres in Sheffield including hospitals, general practices, hospices and other palliative care settings. August 2017 to March 2020, April 2020 to September 2022, and October 2022 to May 2024 were defined as before, during, and after COVID-19 periods, respectively. Absolute percentage-point change in the distribution of organisms was performed at genera level.

**Results:**

A total of 50,827 culture positive isolate episodes from sputum samples were analysed during the study period, of which 25,085 (45.4%; 783.9 isolate episodes per month), 11,644 (22.9%; 388.1 isolate episodes per month) and 14,098 (27.7%; 704.9 isolate episodes per month) were obtained before, during and after COVID-19, respectively. Overall, the commonest cultured genus were *Haemophilus* sp. (21,229/50,827, 41.8%), *Pseudomonas* sp. (5,504/50827, 10.8%), and *Streptococcus* sp. (4,893/50,827, 9.6%). *Haemophilus* sp. remained the commonest cultured genus in each period of the study. *Streptococcus* sp. was the second commonest (2,769/25,085, 11.0%) before COVID-19; fourth commonest (862/11,644, 7.4%) after *Pseudomonas* sp. (1,672/11,644, 14.4%) and Staphylococcus sp. (1,279/11,644, 11.0%) during COVID-19; and third commonest cultured genus (1,262/14098, 9.0%) after *Moraxella* sp. (1,360/14,098, 9.6%) during the post-pandemic period. There were significant variations in the proportion of isolates with Haemophilus sp. (−7.9%, *p* < 0.001) and Pseudomonas sp. (+4.3%, *p* < 0.001) showing the highest change during COVID-19 compared to prior.

**Conclusion:**

We found substantial differences in the number of isolate episodes identified. This could present important considerations for treatment of patients presenting with CAP during future pandemics.

## Introduction

The recent COVID-19 pandemic affected several aspects of healthcare including non-COVID-19 conditions (1, 2). A retrospective study assessing the impact of COVID-19 on non-COVID-19 community-acquired pneumonia showed increases in length of hospital stays and mortality during the pandemic period compared to baseline (3). However, data on the impact of COVID-19 on lower respiratory tract infections in different settings remains scant.

Community acquired pneumonia is a public health concern in both high- and low-income countries and leads to substantial morbidity and mortality (4, 5). According to the 2021 Global Burden of Diseases Study, lower respiratory tract infection (LRTI) including pneumonia and bronchiolitis affected 344 million people worldwide resulting in about 2.2 million deaths, most of whom were children < 5 years and adults >70 years old (6). Pneumonia is the leading infectious causes of death with mortality up to 23% among those admitted to intensive care unit for severe pneumonia (6, 7). In the UK, pneumonia is diagnosed in 5–12% of adults who present to general practitioners with symptoms of LRTI, and up to 14% of those admitted to hospital die (8). In the context of pandemics, mortality attributable to bacterial pneumonia substantially increases (9).

Pandemic-related changes in the epidemiology of organisms responsible for non-COVID-19 pneumonia have been reported (10), and shown to impact patient presentation, mode of care, need for new guidelines, outcome of care, and sequelae of infection (11). Evidence on the effect of COVID-19 on non-COVID-19 pneumonia will inform care at individual level, and preparedness at the heath system level.

In this retrospective study, we aimed to describe temporal changes in organisms recovered from all sputum specimens submitted to the microbiology laboratories at Sheffield Teaching Hospitals before, during, and after the COVID-19 pandemic. These laboratory data were integrated with detailed clinical record review from a randomly selected subset of inpatients admitted with community-acquired pneumonia (CAP) at Sheffield Teaching Hospitals to characterise changes in the epidemiology, demographics, and clinical features of CAP over the same period. Secondary aims were to model predictors of length of hospital stay and in-hospital mortality among patients admitted with CAP before, during, and after COVID-19, in order to better inform empirical antimicrobial treatment at the early presentation of pneumonia.

## Methods

### Study design

This is a retrospective study conducted using anonymised data of patients treated for pneumonia in the greater Sheffield area from 2017 to 2024. This project was registered as a service evaluation with the clinical effectiveness unit of Sheffield Teaching Hospitals NHS Foundation Trust. Reporting follows the Strengthening the Reporting of Observational Studies in Epidemiology guidelines (12).

### Data collection

1. Greater Sheffield data

We collected electronic medical record data on all patient encounters with any health facility from 1st August 2017 to 31st May 2024 within the greater Sheffield area, that resulted in a sputum culture. Information collected included age, date of visit, type of health facility and department, and organism cultured. Health facilities were general practices, acute medical units, and hospital wards. The microbiological unit of analysis was the isolate episode. Each organism identified from a sputum culture was counted separately; therefore, polymicrobial cultures contributed multiple isolate records. Repeat sputum cultures from the same patient within 14 days yielding the same organism were deduplicated into a single isolate episode. August 2017 to March 2020, April 2020 to September 2022, and October 2022 to May 2024 were defined as before, during, and after COVID-19 periods, respectively. We chose March 2020 as the beginning of COVID as this corresponded to the first national lock down period and we observed significant fall in the number of samples sent to our laboratory, these had recovered by October 2022 which was chosen as the end of the acute phase of COVID on UK healthcare for this study.

1. Inpatient cohort data

We compiled a list of every patient admitted to the Infectious Diseases department of Sheffield Teaching Hospitals NHS Foundation Trust with a coded diagnosis of pneumonia between 1st August 2017 and 30th June 2024. We excluded patients who did not have a primary coded diagnosis, and patients with a coded diagnosis which did not represent community acquired pneumonia such as ventilator-associated pneumonia, varicella pneumonitis. Clinical records were reviewed on a case-by-case basis, and those not consistent with a diagnosis of community-acquired pneumonia (e.g., bronchiectasis exacerbation or hospital-acquired pneumonia) were excluded.

We used scanned patient notes to collect data on epidemiological factors such as gender, age at admission, smoking status, asthma diagnosis, chronic obstructive pulmonary disease (COPD) diagnosis and HIV diagnosis. We also collected data on biochemical markers at admission like white cell count, neutrophils, lymphocytes, monocytes, platelets, C-reactive protein (CRP), urea, organism grown in sputum culture, antibiotics administered during admission. Finally, we included prognostic factors at admission such as CURB65 score, as well as length of stay in days, ITU admission, ventilation, outcomes at end of hospital stay (discharge or death), and outcomes at 3 months (remained discharged, died or emergency readmission).

### Main outcome

The primary outcome was the proportion of isolates (at genus level) from sputum samples before, during and after COVID-19. In addition, among the subset of randomly sampled inpatients, we examined the length of hospital stay. Additionally, mortality or emergency admission at 3 months was evaluated as a composite outcome.

### Statistical analysis

Statistical analyses were conducted in Stata (18.5, StataCorp LLC, College Station, TX) and R (version 4.4.2).

Descriptive statistics such as mean and standard deviation; median and interquartile range were used for continuous variables, while frequency and percentage were used for categorical variables.

Variables were compared between the study periods (before COVID-19, during COVID-19, and after COVID-19). Absolute percentage differences between study periods were calculated for all isolates. Z-test for population proportions was used to compare the percentage isolates across any two periods of the study, with the Benjamin-Hochberg procedure employed to control for false discovery rate. To model length of stay from the inpatient cohort data, a negative binomial regression model was fitted due to the variable being count data with overdispersion. Logistic regression was used for the composite dichotomous variable of mortality or emergency admission at 3 months. Both multivariable regression models controlled for age, gender, ventilation, CURB65 and presence of comorbidities. The final logistic regression model contained the study period, age and gender due to the modest event rate. Results from the negative binomial regression are reported as incidence rate ratios (IRR), and results from the logistic regression are reported as odds ratios (OR). Statistical significance was set at *p* < 0.05.

## Results

### Temporal trends in positive sputum cultures

The study duration consisted of 32 months before (August 2017 to March 2020), 30 months during (April 2020 to September 2022), and 20 months after COVID-19 (October 2022 to May 2024). A total of 50,827 microbial isolates recovered from sputum samples were analysed of which 25,085 (45.4%; 783.9 isolate episodes per month), 11,644 (22.9%; 388.1 isolate episodes per month) and 14,098 (27.7%; 704.9 isolate episodes per month) were obtained before, during, and after COVID-19, respectively. Most microbial isolates (37,123/50827, 73.0%) were from outpatient or acute admission settings (Emergency department, Acute medical units, etc). Majority of these (14,494/50827, 28.5%) were obtained from patients aged 65–74 years (Table 1). Detailed species-level data are presented in Supplementary Table S3.

**Table 1:** Characteristics of sputum isolates disaggregated by period relative to COVID-19.

Variables	Total *N* = 50,827	Before COVID-19 *n* = 25,085	During COVID-19 *n* = 11,644	After COVID-19 *n* = 14,098
Location, *n* (%)				
Community^*^	37,123 (73.0)	18,929 (75.5)	7,819 (67.2)	10,375 (73.6)
Hospital	13,704 (27.0)	6,156 (24.5)	3,825 (32.8)	3,723 (26.4)
Age, *n* (%)				
<15	722 (1.4)	365 (1.5)	170 (1.5)	187 (1.3)
15–24	1,563 (3.1)	729 (2.9)	359 (3.1)	475 (3.4)
25–44	5,266 (10.4)	2,430 (9.7)	1,145 (9.8)	1,691 (12.0)
45–64	13,757 (27.1)	6,684 (26.6)	3,141 (27.0)	3,932 (27.9)
65–74	14,494 (28.5)	7,484 (29.8)	3,350 (28.8)	3,660 (26.0)
75–84	11,948 (23.5)	5,838 (23.3)	2,829 (24.3)	3,281 (23.3)
85+	3,077 (6.1)	1,555 (6.2)	650 (5.6)	872 (6.2)
Mean (SD)	63.9 (17.8)	64.2 (17.6)	64.1 (17.7)	63.1 (18.2)
Isolates, *n* (%)				
Aspergillus sp.	287 (0.6)	84 (0.3)	114 (1.0)	89 (0.6)
Candida sp.	1,074 (2.1)	217 (0.9)	477 (4.1)	380 (2.7)
Corynebacterium sp.	554 (1.1)	248 (1.0)	176 (1.5)	130 (0.9)
Enterobacter sp.	455 (0.9)	214 (0.9)	119 (1.0)	122 (0.9)
Escherichia sp.	1898 (3.7)	858 (3.4)	577 (5.0)	463 (3.3)
Haemophilus sp.	21,229 (41.8)	10,896 (43.4)	4,143 (35.6)	6,190 (43.9)
Klebsiella sp.	1,592 (3.1)	715 (2.9)	405 (3.5)	472 (3.3)
Moraxella sp.	4,655 (9.2)	2,505 (10.0)	790 (6.8)	1,360 (9.6)
Proteus sp.	566 (1.1)	239 (1.0)	151 (1.3)	176 (1.2)
Pseudomonas sp.	5,504 (10.8)	2,532 (10.1)	1,672 (14.4)	1,300 (9.2)
Serratia sp.	997 (2.0)	421 (1.7)	249 (2.1)	327 (2.3)
Staphylococcus sp.	4,484 (8.8)	2038 (8.1)	1,279 (11.0)	1,167 (8.3)
Stenotrophomonas sp.	589 (1.2)	318 (1.3)	145 (1.2)	126 (0.9)
Streptococcus sp.	4,893 (9.6)	2,769 (11.0)	862 (7.4)	1,262 (9.0)
Other	2050 (4.0)	1,031 (4.1)	485 (4.2)	534 (3.8)

*N*, total number; *n*, subtotal; SD, standard deviation; sp., species.

**Figure 1:**
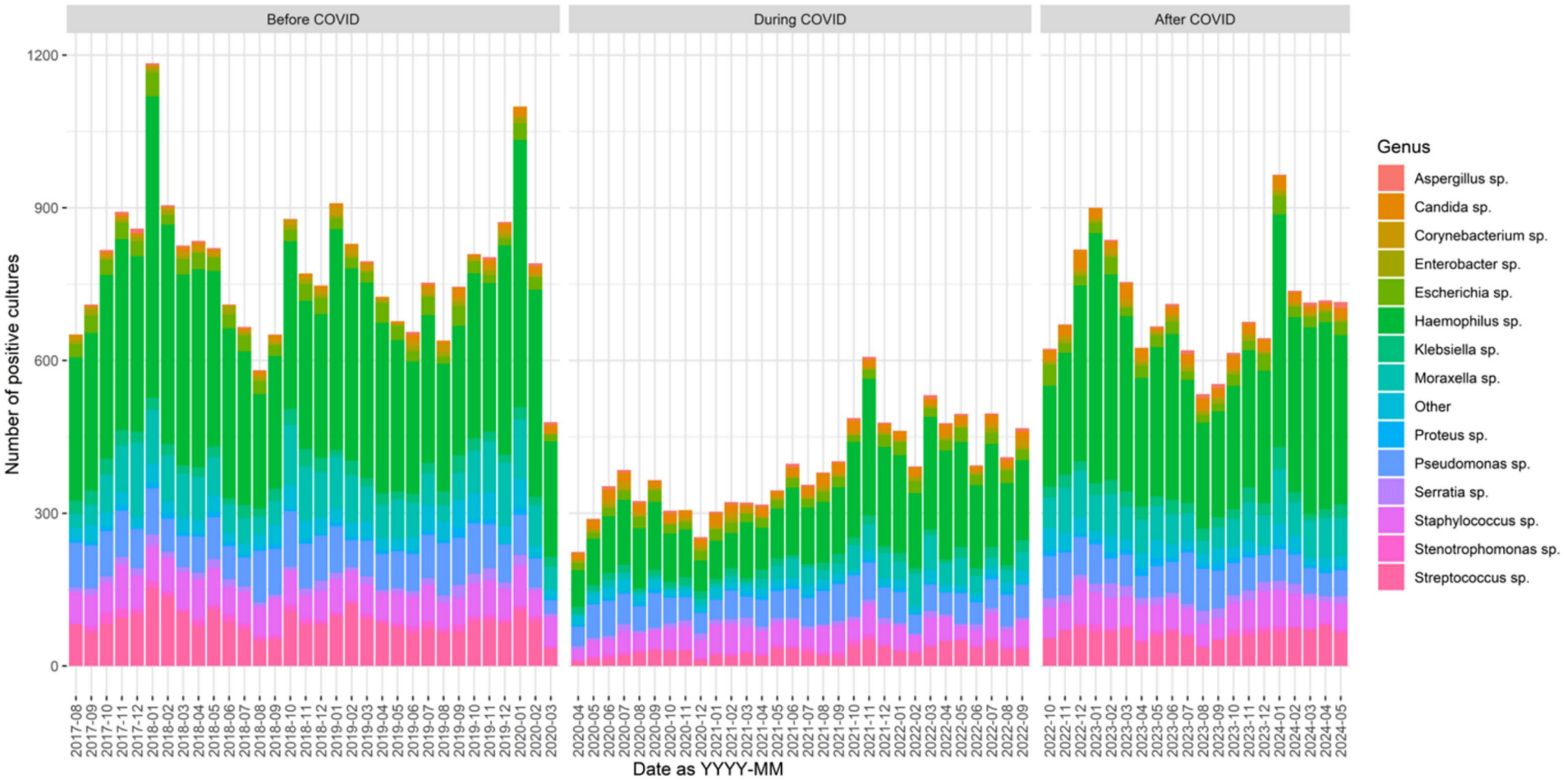
Monthly number of culture-positive organisms recovered from sputum samples from 2017 to 2024.

Overall, the mean monthly number of positive isolates was 784 before COVID-19, 388 during COVID-19, and 705 after COVID-19 (Figure 1). Across the entire study duration, *Haemophilus* sp. (21,229/50,827, 41.8%) was the most frequently cultured genus, follow by *Pseudomonas* sp. (5,504/50,827, 10.8%) and *Streptococcus* sp. (4,893/50,827, 9.6%) as the second and third most frequently cultured genera.

*Haemophilus* sp. remained the most frequently cultured genus in each period of the study. *Streptococcus* sp. was the second commonest (2,769/25,085, 11.0%) before COVID-19; fourth commonest (862/11,644, 7.4%) after *Pseudomonas* sp. (1,672/11,644, 14.4%) and *Staphylococcus* sp. (1,279/11,644, 11.0%) during COVID-19; and third commonest cultured genus (1,262/14,098, 9.0%) after *Moraxella* sp. (1,360/14,098, 9.6%) during the post-pandemic period. Detailed species-level data for Streptococcus sp. are presented in Supplementary Table S4.

In the outpatient or acute settings *Haemophilus* sp. (18,420/37,123, 49.6%), *Streptococcus* sp. (4,288/37,123, 11.6%) and *Moraxella* sp. (4,131/37,123, 11.1%) were the top three identified genera. In these settings, *Streptococcus* sp. (2,427/18,929, 12.8%) and *Moraxella* sp. (2,210/18,929, 11.7%) remained the second and third most cultured genera before COVID-19. During COVID-19, the second and third most cultured genera were *Pseudomonas* sp. (966/7819, 12.4%) and *Staphylococcus* sp. (792/7819, 10.1%). After COVID-19, the second and third most cultured genera were *Moraxella* sp. (1,219/10,375, 11.7%) and *Streptococcus* sp. (1,122/10,375, 10.8%) (Supplementary Figure S1; Supplementary Table S1).

For inpatient settings, the top three genera were *Haemophilus* sp. (2,809/13,704, 20.5%), *Pseudomonas* sp. (2,232/13,704, 16.3%) and *Staphylococcus* sp. (1,484/13,704, 10.8%). Thus, while Haemophilus sp. remained the most cultured, its proportion substantially decreased compared to outpatient or acute settings. The most frequently cultured genus in inpatient settings during COVID-19 was *Pseudomonas* sp. (706/3825, 18.5%). Haemophilus sp. was once again most cultured after COVID-19 (Supplementary Figure S1b; Supplementary Table S2).

**Figure 2:**
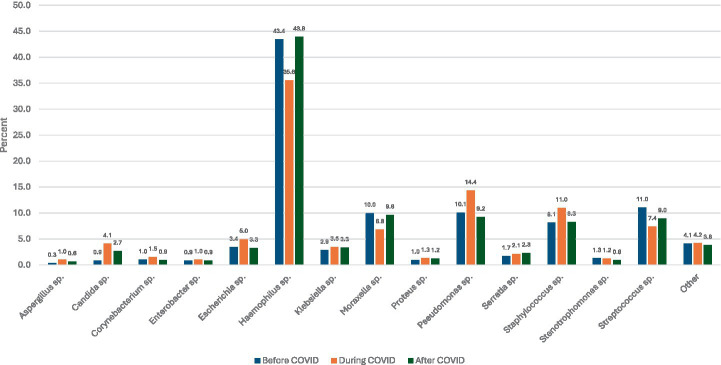
Proportion of isolates for each study period.

Figure 2 shows the proportion of each genus before, during and after COVID-19. The proportion of *Aspergillus* sp. and *Candida* sp. cultured during COVID-19 compared to before the pandemic significantly increased by 0.7% (*p* < 0.001) and 3.2% (*p* < 0.001) respectively. Streptococcus sp. decreased by 3.6% (*p* < 0.001), Moraxella sp. decreased by 3.2% (*p* < 0.001) and *Haemophilus* sp. decreased by 7.9% (*p* < 0.001) during COVID-19 compared to before COVID-19. Detailed percent differences during each period are presented on Table 2. Additional data on proportional differences at species-level for Streptococcus sp. are presented in Supplementary Table S5.

**Table 2:** Percent difference of sputum isolates during each period of the study.

Isolates	During COVID vs Before COVID		After COVID vs During COVID		After COVID vs Before COVID	
	% difference	*p value*	% difference	*p value*	% difference	*p value*
Aspergillus sp.	0.7	<0.001	−0.3	0.002	0.3	<0.001
Candida sp.	3.2	<0.001	−1.4	<0.001	1.8	<0.001
Corynebacterium sp.	0.5	<0.001	−0.6	<0.001	−0.1	0.553
Enterobacter sp.	0.2	0.126	−0.2	0.217	0.0	0.945
Escherichia sp.	1.5	<0.001	−1.7	<0.001	−0.1	0.492
Haemophilus sp.	−7.9	<0.001	8.3	<0.001	0.5	0.373
Klebsiella sp.	0.6	0.001	−0.1	0.590	0.5	0.006
Moraxella sp.	−3.2	<0.001	2.9	<0.001	−0.3	0.288
Proteus sp.	0.3	0.003	0.0	0.772	0.3	0.007
Pseudomonas sp.	4.3	<0.001	−5.1	<0.001	−0.9	0.006
Serratia sp.	0.5	0.002	0.2	0.350	0.6	<0.001
Staphylococcus sp.	2.9	<0.001	−2.7	<0.001	0.2	0.608
Stenotrophomonas sp.	0.0	0.897	−0.4	0.007	−0.4	<0.001
Streptococcus sp.	−3.6	<0.001	1.5	<0.001	−2.1	<0.001
Other	0.1	0.826	−0.4	0.130	−0.3	0.124

### Clinical characteristics of inpatients with community acquired pneumonia

We characterised the demographic and clinical characteristics of randomly selected inpatients admitted with community-acquired pneumonia (Table 3). A total of 399 patients were analysed: 168 (42.1%) before COVID-19, 114 (28.6%) during COVID-19, and 117 (29.3%) after COVID-19. Of the 399 patients, 214 (53.6%) were male. The mean age was 49.3 years (standard deviation: 18.3). CURB-65 scores of 2 and above were seen in 11/168 (6.5%), 27/114 (23.7%) and 15/117 (12.8%) patients before, during and after COVID-19, respectively.

**Table 3:** Demographic and clinical characteristics of randomly selected inpatients admitted with community-acquired pneumonia.

Variables	Total *N* = 399	Before COVID *n* = 168	During COVID *n* = 114	After COVID *n* = 117
Gender, *n* (%)				
Female	185(46.4)	81 (48.2)	42 (36.8)	62 (53.0)
Male	214(53.6)	87 (51.8)	72 (63.2)	55 (47.0)
Age				
Mean (SD)	49.3 (18.3)	46.3 (16.9)	56.9 (17.9)	46.2 (18.6)
ITU/CC admission, *n* (%)				
No	388(97.2)	166 (98.8)	108 (94.7)	114 (97.4)
Yes	11(2.8)	2 (1.2)	6 (5.3)	3 (2.6)
Comorbidities, *n* (%)				
No	74(18.5)	36 (21.4)	6 (5.3)	32 (27.4)
Yes	325(81.5)	132 (78.6)	108 (94.7)	85 (72.6)
CURB 65 score, *n* (%)				
0	251 (62.9)	120 (71.4)	52 (45.6)	79 (67.5)
1	91 (22.8)	33 (19.6)	35 (30.7)	23 (19.7)
2	47 (11.8)	11 (6.5)	23 (20.2)	13 (11.1)
3	6 (1.5)	0 (0)	4 (3.5)	2 (1.7)
Missing	4 (1.0)	4 (2.4)	0 (0)	0 (0)
Smoking				
Never	193 (48.4)	79 (47.0)	47 (41.2)	67 (57.3)
Former	87 (21.8)	36 (21.4)	27 (23.7)	24 (20.5)
Current	98 (24.6)	43 (25.6)	34 (29.8)	21 (17.9)
Missing	21 (5.3)	10 (6.0)	6 (5.3)	5 (4.3)
WBC x10^3^ /uL, mean (SD)				
Total	12.4 (6.0)	11.9 (5.9)	13.7 (6.6)	11.9 (5.3)
Neutrophil	10.0 (5.7)	9.42 (5.6)	11.4 (6.3)	9.50 (5.0)
Monocyte	0.86 (0.44)	0.88 (0.46)	0.88 (0.47)	0.825 (0.37)
Lymphocyte	1.40 (0.91)	1.47 (0.862)	1.28 (1.06)	1.43 (0.786)
Platelets				
Mean (SD)	276 (130)	291 (148)	255 (115)	276 (115)
Urea				
Mean (SD)	5.6 (3.3)	5.2 (2.8)	6.5 (4.4)	5.3 (2.8)
CRP				
Mean (SD)	141.6 (113.9)	127.6 (111.0)	140.3 (119.5)	163.0 (110.1)
Outcome, *n* (%)				
Discharged	390 (97.7)	167 (99.4)	109 (95.6)	114 (97.4)
Died	9 (2.3)	1 (0.6)	5 (4.4)	3 (2.6)
Outcome at 3 months, *n* (%)				
Death/emergency admission	18 (4.5)	3 (1.8)	10 (8.8)	5 (4.3)
No	381 (95.5)	165 (98.2)	104 (91.2)	112 (95.7)

STH, Sheffield Teaching Hospitals NHS Foundation Trust; SD, standard deviation; ITU/CC, intensive therapy/critical care unit; WBC, white blood cells; CRP, c-reactive protein.

**Figure 3:**
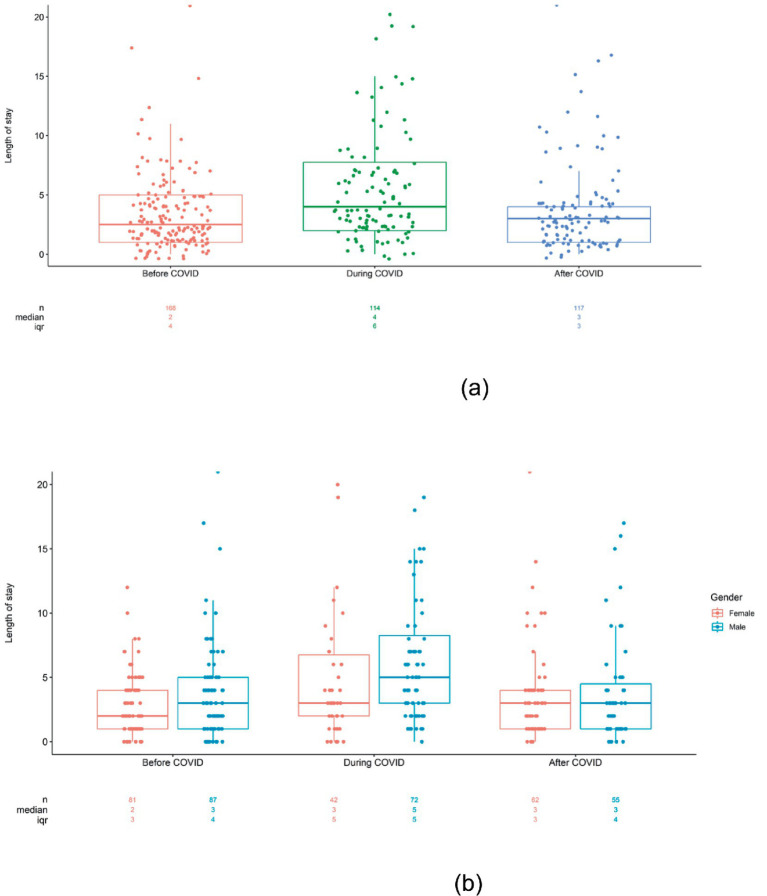
Length of hospital stay **(a)** before, during and after COVID-19; and **(b)** during the study periods disaggregated by gender.

Mortality or emergency admission within 3 months of discharge was recorded in 3/168 (1.8%), 10/114 (8.8%) and 5/117 (4.3%) patients before, during and after COVID-19, respectively. Figure 3A shows the length of hospital stay during the study periods. Overall, patients were in hospital for a minimum of 0 days to a maximum of 52 days, with a median of 3 days (interquartile range [IQR], 1–5). Before COVID-19, the maximum length of stay was 47 days, median 2.5 (IQR: 1–5); during COVID-19 patients were hospitalised up to 52 days, median 4 (IQR: 2–8); and after COVID-19 patients were hospitalised up to 22 days, median 3 (IQR: 1–4). Median length of hospital stay was higher in males (Figure 3B).

**Figure 4:**
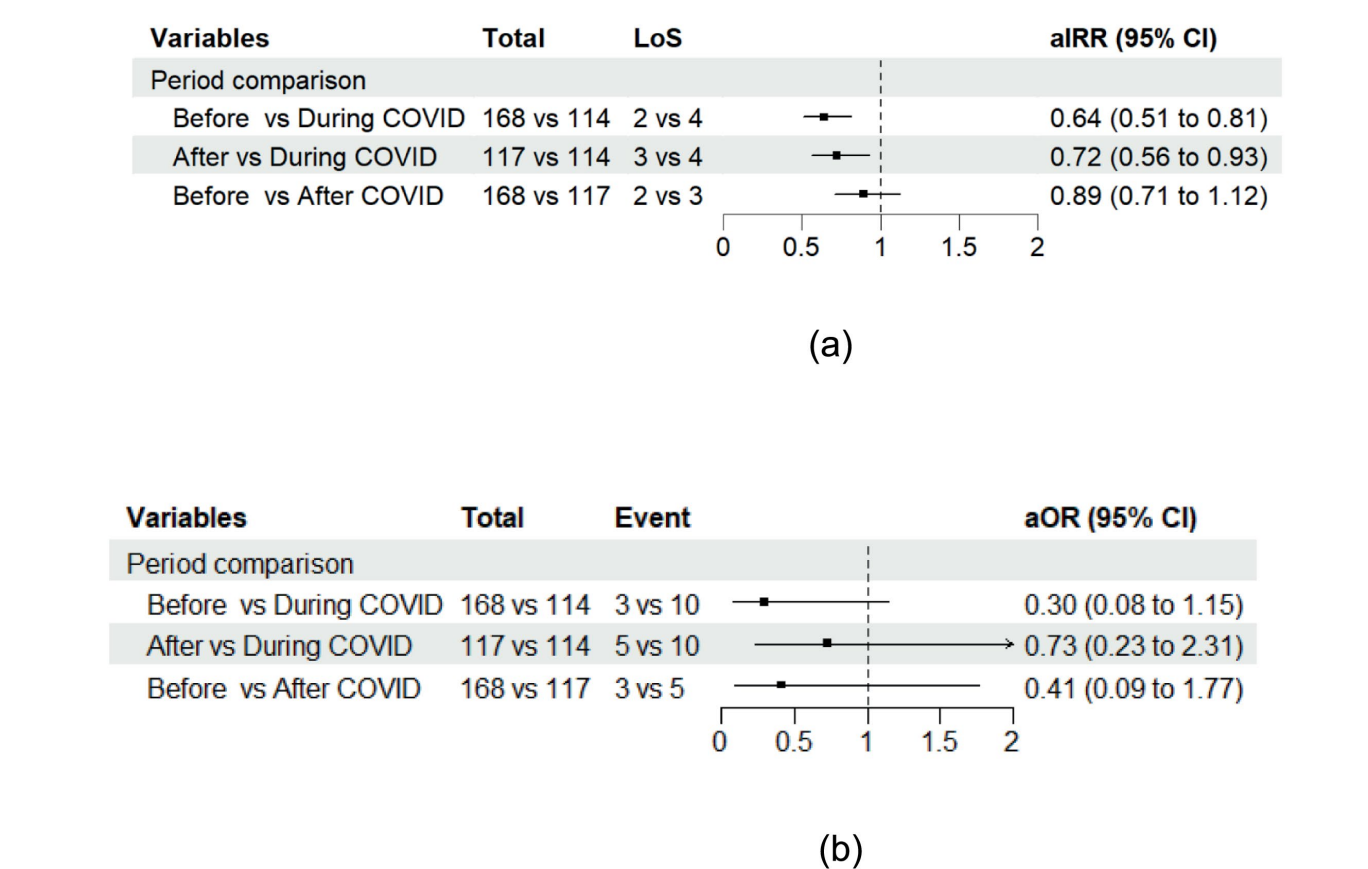
**(a)** Negative binomial regression for length of hospital stay, adjusted for age, gender, ventilation, CURB-65 and presence of comorbidities. *LoS, length of stay (days); aIRR, adjusted incidence rate ratio.* **(b)** Logistic regression for mortality or emergency admission within 3 months of discharge, adjusted for study period, age and gender *aOR, adjusted odds ratio.*

The risk of longer hospital stays was lower before COVID-19 (aIRR, 0.66, CI: 0.59–0.73, *p* < 0.001) and after COVID-19 (aIRR, 0.70, CI: 0.62–0.78, *p* < 0.001) compared to during COVID-19. However, there was no statistically significant difference in length of hospital stay between the period before COVID-19 and after COVID-19 (see Figure 4A). Furthermore, there was no statistically significant difference between any study period in terms of mortality or emergency admission within 3 months of discharge (see Figure 4B).

## Discussion

We conducted a study to determine prevalence of pathogens isolated from respiratory samples sent to the clinical microbiology laboratory before, during and after COVID-19. We also described the demographics and clinical characteristics in a subset of patients admitted with pneumonia and modelled the predictors of length of hospital stay and mortality, before, during, and after COVID-19. *Haemophilus* sp. was the commonest cultured genus before, during and after COVID-19. *Aspergillus* sp. and *Candida* sp. were the only organisms with proportional increases during COVID-19 compared to before COVID-19. There were significant variations in the proportion of organisms cultured during the study periods, as well as difference in-between care settings (inpatient, community or acute care). The median length of hospital stay was higher during COVID-19 compared to before or after COVID. However, there was no difference in length of hospital before COVID-19 vs. after COVID-19.

Overall, the commonest organism cultured in our study was *Haemophilus* sp. This was highest before COVID-19, declined significantly during the pandemic and began to rise after the pandemic without reaching pre-pandemic levels. In keeping with findings in our work, other studies reported a high rate of infections with *Haemophilus* sp. in the period before COVID-19, which declined steeply during COVID-19 and gradually increased in the post pandemic period (13, 14). Globally, there were changes in the clinical epidemiology of pathogens where invasive infections with organisms like *Haemophilus* sp. were noted to have declined during the pandemic period (15, 16). In our study population, the mean age of patients undergoing sputum culture was 63.9 years, with nearly 60% being 65 years and older. The burden of chronic obstructive pulmonary disease (COPD) exponentially increases with age, and more than quadruples among people above 65 years compared to those below 40 years (17, 18). In the presence of COPD, *Haemophilus* sp. has been reported as the commonest bacterial cause of lung infection (19–21). These could explain the overall high prevalence of *Haemophilus* sp. observed in our study. The reduction observed during COVID-19 could be due to decreased admissions with people not seeking healthcare. Furthermore, there were reduced number of samples sent during this period due to general practitioners treating empirically with antibiotics.

We found *Aspergillus* sp. and *Candida* sp. to be the only organisms with relative increases in fungal isolates in sputum cultures during COVID-19 compared to their pre-pandemic prevalence. However, this likely represent changes in colonising flora rather than invasive fungal infection and could be secondary to increased empirical antibiotic use both in primary and secondary care (22), as well as reduced admission rates. A similar pattern has been reported elsewhere, that fungal infections were observed to substantially increase during COVID-19 (23). Although exact pathogenesis is poorly understood, it has been suggested that the presence of SARS-CoV-2 itself, immunosuppressive therapies, previous antibiotics use and underlying chronic pulmonary disease potentially facilitate the increase in fungal infections during COVID (24). Although Candida in respiratory specimens often represents non-pathogenic colonising flora (25), there was a strong association between COVID-19 and invasive aspergillois (26).

Inpatients to the Infectious Diseases unit were more likely to stay in hospital longer during the pandemic compared to the before and after periods. This result may be due to several factors related to operational delays and case complexity. Operational pressures in the pandemic included staffing shortages through COVID-19 illness, COVID-19 testing limitations, care resource allocation, patient transport logistics and off-site institutional bed capacity (care home, rehabilitation unit, intermediate care unit) may contribute to delays discharging patients who are medically ready for discharge. Our findings suggest pre-pandemic planning is needed at local level to ensure bed flow disruption and risk of poorer outcomes for patients is minimized. There were no significant differences in length of hospital stay for patients before the pandemic compared to after the pandemic. This suggests easing of operational pressures post-pandemic had an influence on length of hospital stay for patients with CAP. Further investigation into whether COVID-19 co-infection during the pandemic contributed to longer hospital stays for these inpatients with Community Acquired Pneumonia may yield interesting findings, but this goes beyond the scope of this study. Current evidence does suggest that COVID-19 co-infection does increase hospital length of stay (27).

Interestingly, we found no significant differences in mortality or emergency readmission at 3 months for patients with CAP before, during and after the pandemic. We did not expect these results given well established associations between hospital capacity strain and inpatient outcomes (28). Our findings are consistent with a similar retrospective study which found no significant mortality changes in non-COVID-19 conditions before and during the pandemic (1). Our inpatient cohort included patients admitted to a specialist Infectious Disease unit, where management by infection-focused specialists may have influenced patient outcomes. However, it is possible the cohort of inpatients used for this study may not be representative of the wider inpatient population within the trust at the time. Our cohort had a mean age of 49.3, and 18.5% of patients had no comorbidities documented. Further retrospective studies could use inpatient cohorts with a primary CAP diagnosis in other hospital departments such as Frailty and General Medical Units.

## Conclusion

In this study, the commonest isolated organisms before, during and after COVID-19, were *Haemophilus* sp., *Pseudomonas* sp. and *Streptococcus* sp. There were significant variations in the proportion of the same organism across the study periods with a higher proportion of fungal isolate during COVID-19 compared to prior. Additionally, length of stay was longer during the pandemic, but we did not detect any statistically significant differences in mortality or the number of emergency readmission caused by the pandemic.

Our findings present important considerations for treatment of patients presenting with pneumonia during prolonged service disruptions and emphasize the importance of continuous update of local surveillance data to ensure changes in the trend of organisms are captured.

## Data Availability

The raw data supporting the conclusions of this article will be made available by the authors, without undue reservation.
