# Supplementary material for: Impact of COVID-19 on sputum isolates and hospital outcomes among patients with pneumonia in Sheffield, United Kingdom: a retrospective cohort study
Source: Front Public Health. 2026 Jun 30;14:1816789. doi: 10.3389/fpubh.2026.1816789 (PMC13364976; doi:10.3389/fpubh.2026.1816789)
Supplement: Supplementary file 1 [file Supplementary_file_1.DOCX]

**Supplementary material**

**Impact of COVID-19 on sputum isolates and hospital outcomes among patients with pneumonia in Sheffield, United Kingdom: a retrospective cohort study**


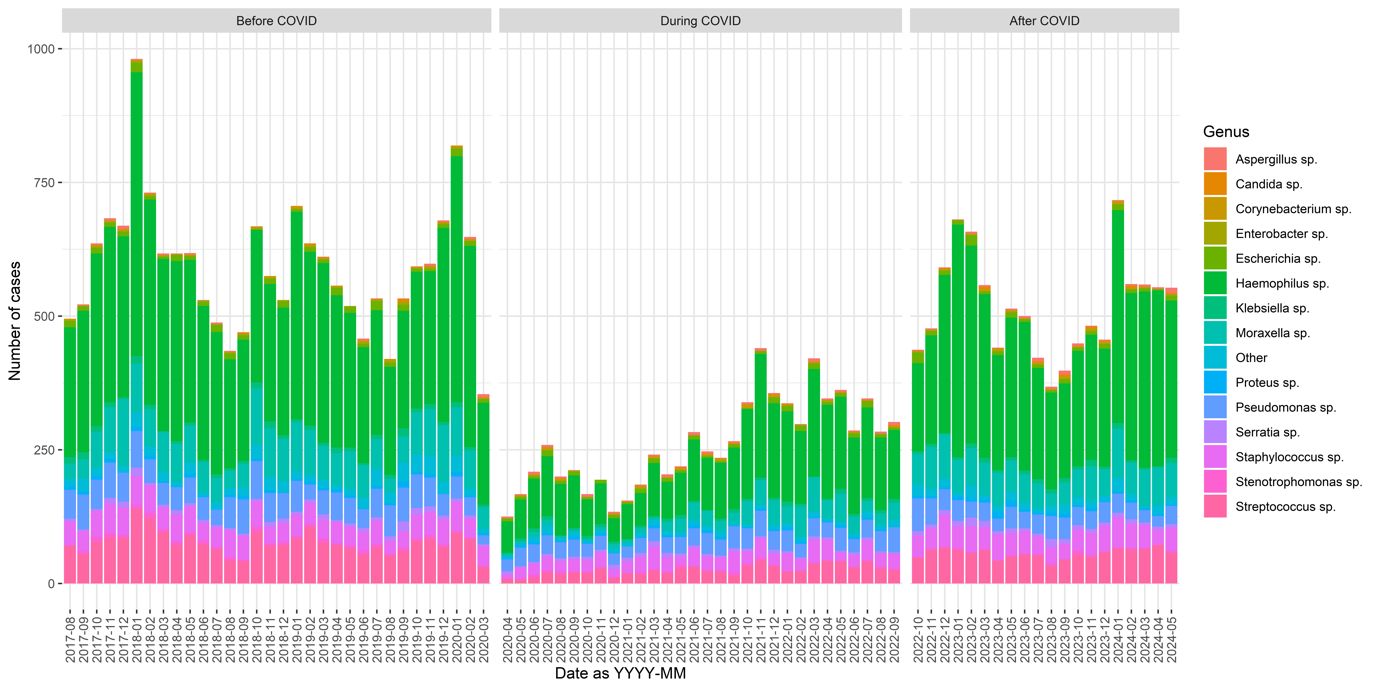


(a)


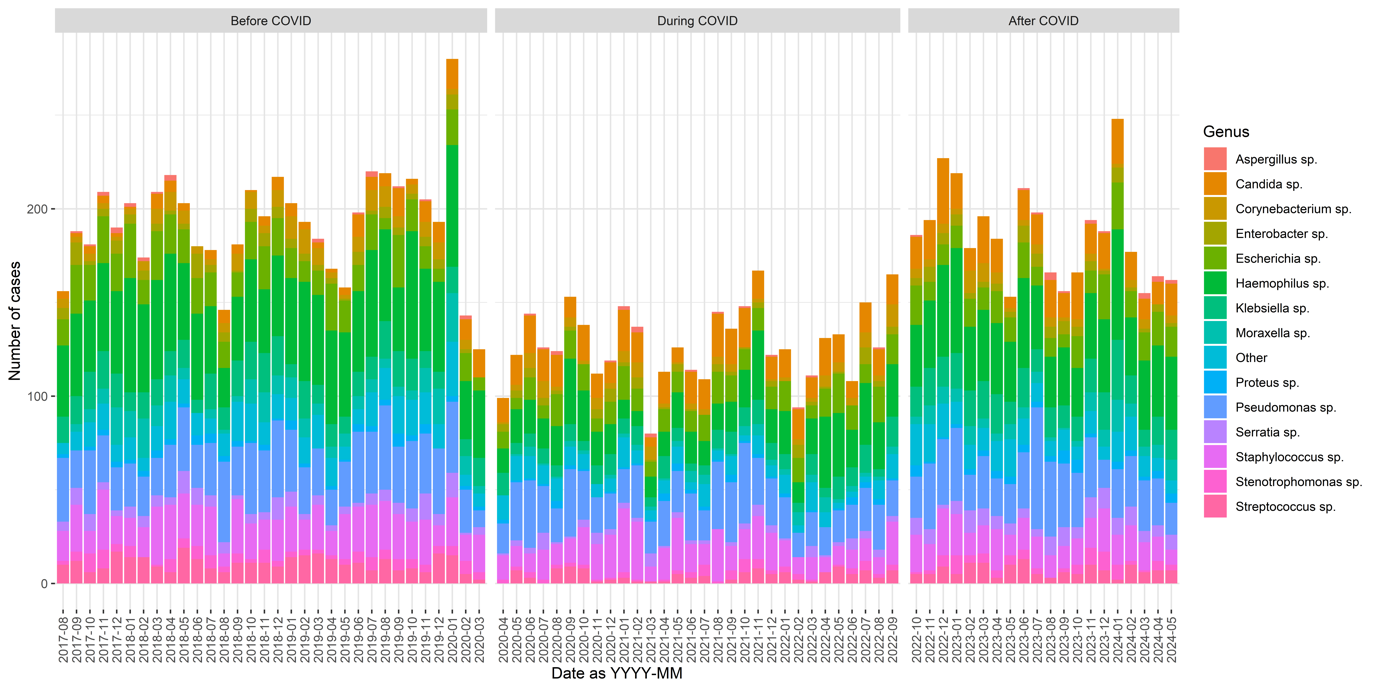


(b)

Figure S1. Monthly number of culture positive isolates (at genus level) from 2017 to 2024 in (a) outpatient and acute settings, (b) inpatient settings.

Figure S2. Relative proportions of isolates during the pre-pandemic, pandemic and post-pandemic periods.

Table S1. Characteristics of sputum isolates in outpatient and acute settings, disaggregated by period relative to COVID-19.

| **Variables** | **Total**  **N=37123** | **Before COVID n=18929** | **During COVID n=7819** | **After COVID n=10375** |
| --- | --- | --- | --- | --- |
| Age |  |  |  |  |
| <15 | 547 (1.5) | 281 (1.5) | 122 (1.6) | 144 (1.4) |
| 15 - 24 | 1167 (3.1) | 506 (2.7) | 254 (3.2) | 407 (3.9) |
| 25 - 44 | 3792 (10.2) | 1830 (9.7) | 692 (8.9) | 1270 (12.2) |
| 45 - 64 | 9850 (26.5) | 5056 (26.7) | 1943 (24.8) | 2851 (27.5) |
| 65 - 74 | 10822 (29.2) | 5821 (30.8) | 2344 (30.0) | 2657 (25.6) |
| 75 - 84 | 8806 (23.7) | 4359 (23.0) | 2024 (25.9) | 2423 (23.4) |
| 85+ | 2139 (5.8) | 1076 (5.7) | 440 (5.6) | 623 (6.0) |
| Mean (SD) | 64.0 (17.7) | 64.3 (17.3) | 64.8 (17.6) | 62.9 (18.5) |
| Isolates |  |  |  |  |
| Aspergillus sp. | 224 (0.6) | 59 (0.3) | 95 (1.2) | 70 (0.7) |
| Candida sp. | 64 (0.2) | 24 (0.1) | 17 (0.2) | 23 (0.2) |
| Corynebacterium sp. | 145 (0.4) | 50 (0.3) | 51 (0.7) | 44 (0.4) |
| Enterobacter sp. | 120 (0.3) | 63 (0.3) | 31 (0.4) | 26 (0.3) |
| Escherichia sp. | 655 (1.8) | 294 (1.6) | 196 (2.5) | 165 (1.6) |
| Haemophilus sp. | 18420 (49.6) | 9456 (50.0) | 3534 (45.2) | 5430 (52.3) |
| Klebsiella sp. | 518 (1.4) | 248 (1.3) | 140 (1.8) | 130 (1.3) |
| Moraxella sp. | 4131 (11.1) | 2210 (11.7) | 702 (9.0) | 1219 (11.7) |
| Proteus sp. | 359 (1.0) | 152 (0.8) | 99 (1.3) | 108 (1.0) |
| Pseudomonas sp. | 3273 (8.8) | 1593 (8.4) | 966 (12.4) | 714 (6.9) |
| Serratia sp. | 552 (1.5) | 225 (1.2) | 156 (2.0) | 171 (1.6) |
| Staphylococcus sp. | 3000 (8.1) | 1364 (7.2) | 792 (10.1) | 844 (8.1) |
| Stenotrophomonas sp. | 275 (0.7) | 168 (0.9) | 71 (0.9) | 36 (0.3) |
| Streptococcus sp. | 4288 (11.6) | 2427 (12.8) | 739 (9.5) | 1122 (10.8) |
| Other | 1099 (3.0) | 596 (3.1) | 230 (2.9) | 273 (2.6) |

Table S2. Characteristics of sputum isolates in inpatient settings, disaggregated by period relative to COVID-19.

| **Variables** | **Total**  **N=13704** | **Before COVID n=6156** | **During COVID n=3825** | **After COVID n=3723** |
| --- | --- | --- | --- | --- |
| Age |  |  |  |  |
| <15 | 175 (1.3) | 84 (1.4) | 48 (1.3) | 43 (1.2) |
| 15 - 24 | 396 (2.9) | 223 (3.6) | 105 (2.7) | 68 (1.8) |
| 25 - 44 | 1474 (10.8) | 600 (9.7) | 453 (11.8) | 421 (11.3) |
| 45 - 64 | 3907 (28.5) | 1628 (26.4) | 1198 (31.3) | 1081 (29.0) |
| 65 - 74 | 3672 (26.8) | 1663 (27.0) | 1006 (26.3) | 1003 (26.9) |
| 75 - 84 | 3142 (22.9) | 1479 (24.0) | 805 (21.0) | 858 (23.0) |
| 85+ | 938 (6.8) | 479 (7.8) | 210 (5.5) | 249 (6.7) |
| Mean (SD) | 63.7 (18.0) | 64.2 (18.6) | 62.6 (17.6) | 64.0 (17.3) |
| Isolates |  |  |  |  |
| Aspergillus sp. | 63 (0.5) | 25 (0.4) | 19 (0.5) | 19 (0.5) |
| Candida sp. | 1010 (7.4) | 193 (3.1) | 460 (12.0) | 357 (9.6) |
| Corynebacterium sp. | 409 (3.0) | 198 (3.2) | 125 (3.3) | 86 (2.3) |
| Enterobacter sp. | 335 (2.4) | 151 (2.5) | 88 (2.3) | 96 (2.6) |
| Escherichia sp. | 1243 (9.1) | 564 (9.2) | 381 (10.0) | 298 (8.0) |
| Haemophilus sp. | 2809 (20.5) | 1440 (23.4) | 609 (15.9) | 760 (20.4) |
| Klebsiella sp. | 1074 (7.8) | 467 (7.6) | 265 (6.9) | 342 (9.2) |
| Moraxella sp. | 524 (3.8) | 295 (4.8) | 88 (2.3) | 141 (3.8) |
| Proteus sp. | 207 (1.5) | 87 (1.4) | 52 (1.4) | 68 (1.8) |
| Pseudomonas sp. | 2231 (16.3) | 939 (15.3) | 706 (18.5) | 586 (15.7) |
| Serratia sp. | 445 (3.2) | 196 (3.2) | 93 (2.4) | 156 (4.2) |
| Staphylococcus sp. | 1484 (10.8) | 674 (10.9) | 487 (12.7) | 323 (8.7) |
| Stenotrophomonas sp. | 314 (2.3) | 150 (2.4) | 74 (1.9) | 90 (2.4) |
| Streptococcus sp. | 605 (4.4) | 342 (5.6) | 123 (3.2) | 140 (3.8) |
| Other | 951 (6.9) | 435 (7.1) | 255 (6.7) | 261 (7.0) |

Table S3. Sputum isolates at species level

| **Isolates** | **Before COVID (N=25085)** | **During COVID (N=11644)** | **After COVID (N=14098)** | **Overall (N=50827)** |
| --- | --- | --- | --- | --- |
| Achromobacter denitrificans | 1 (0.0%) | 0 (0%) | 0 (0%) | 1 (0.0%) |
| Achromobacter sp. | 1 (0.0%) | 2 (0.0%) | 0 (0%) | 3 (0.0%) |
| Achromobacter xylosoxidans | 20 (0.1%) | 11 (0.1%) | 8 (0.1%) | 39 (0.1%) |
| Acinetobacter baumanii | 17 (0.1%) | 11 (0.1%) | 23 (0.2%) | 51 (0.1%) |
| Acinetobacter baylyi | 3 (0.0%) | 0 (0%) | 0 (0%) | 3 (0.0%) |
| Acinetobacter calcoaceticus | 1 (0.0%) | 2 (0.0%) | 0 (0%) | 3 (0.0%) |
| Acinetobacter guillouiae | 1 (0.0%) | 0 (0%) | 0 (0%) | 1 (0.0%) |
| Acinetobacter haemolyticus | 2 (0.0%) | 0 (0%) | 0 (0%) | 2 (0.0%) |
| Acinetobacter junii | 2 (0.0%) | 0 (0%) | 0 (0%) | 2 (0.0%) |
| Acinetobacter lwoffi | 3 (0.0%) | 0 (0%) | 1 (0.0%) | 4 (0.0%) |
| Acinetobacter nosocomialis | 1 (0.0%) | 0 (0%) | 0 (0%) | 1 (0.0%) |
| Acinetobacter pittii | 9 (0.0%) | 3 (0.0%) | 4 (0.0%) | 16 (0.0%) |
| Acinetobacter proteolyticus | 2 (0.0%) | 1 (0.0%) | 0 (0%) | 3 (0.0%) |
| Acinetobacter radioresistens | 1 (0.0%) | 0 (0%) | 0 (0%) | 1 (0.0%) |
| Acinetobacter sp. | 11 (0.0%) | 5 (0.0%) | 1 (0.0%) | 17 (0.0%) |
| Acinetobacter tjernbergiae | 1 (0.0%) | 0 (0%) | 0 (0%) | 1 (0.0%) |
| Acinetobacter ursingii | 1 (0.0%) | 1 (0.0%) | 2 (0.0%) | 4 (0.0%) |
| Actinobacillus sp. | 3 (0.0%) | 1 (0.0%) | 2 (0.0%) | 6 (0.0%) |
| Actinomyces odontolyticus | 1 (0.0%) | 0 (0%) | 0 (0%) | 1 (0.0%) |
| Aeromonas hydrophila | 1 (0.0%) | 0 (0%) | 0 (0%) | 1 (0.0%) |
| Aeromonas sp. | 2 (0.0%) | 1 (0.0%) | 1 (0.0%) | 4 (0.0%) |
| Aggregatibacter segnis | 13 (0.1%) | 1 (0.0%) | 2 (0.0%) | 16 (0.0%) |
| Arcanobacterium haemolyticum | 1 (0.0%) | 0 (0%) | 0 (0%) | 1 (0.0%) |
| Aspergillus fumigatus | 8 (0.0%) | 30 (0.3%) | 52 (0.4%) | 90 (0.2%) |
| Aspergillus niger | 1 (0.0%) | 10 (0.1%) | 9 (0.1%) | 20 (0.0%) |
| Aspergillus sp. | 75 (0.3%) | 69 (0.6%) | 23 (0.2%) | 167 (0.3%) |
| Bacillus cereus | 2 (0.0%) | 0 (0%) | 0 (0%) | 2 (0.0%) |
| Bacillus circulans | 1 (0.0%) | 1 (0.0%) | 0 (0%) | 2 (0.0%) |
| Bacillus sp. | 1 (0.0%) | 0 (0%) | 0 (0%) | 1 (0.0%) |
| Bergeyella zoohelcum | 1 (0.0%) | 0 (0%) | 0 (0%) | 1 (0.0%) |
| Bordetella bronchiseptica | 3 (0.0%) | 1 (0.0%) | 0 (0%) | 4 (0.0%) |
| Burkholderia cenocepacia | 1 (0.0%) | 0 (0%) | 0 (0%) | 1 (0.0%) |
| Burkholderia cepacia | 1 (0.0%) | 0 (0%) | 0 (0%) | 1 (0.0%) |
| Burkholderia cepacia complex | 2 (0.0%) | 4 (0.0%) | 1 (0.0%) | 7 (0.0%) |
| Burkholderia gladioli | 2 (0.0%) | 2 (0.0%) | 5 (0.0%) | 9 (0.0%) |
| Candida albicans | 157 (0.6%) | 337 (2.9%) | 257 (1.8%) | 751 (1.5%) |
| Candida catenulata | 1 (0.0%) | 0 (0%) | 0 (0%) | 1 (0.0%) |
| Candida dubliniensis | 4 (0.0%) | 19 (0.2%) | 37 (0.3%) | 60 (0.1%) |
| Candida glabrata | 26 (0.1%) | 51 (0.4%) | 47 (0.3%) | 124 (0.2%) |
| Candida kefyr | 1 (0.0%) | 7 (0.1%) | 1 (0.0%) | 9 (0.0%) |
| Candida krusei | 3 (0.0%) | 14 (0.1%) | 2 (0.0%) | 19 (0.0%) |
| Candida lambica | 1 (0.0%) | 0 (0%) | 0 (0%) | 1 (0.0%) |
| Candida lusitaniae | 2 (0.0%) | 6 (0.1%) | 3 (0.0%) | 11 (0.0%) |
| Candida parapsilosis | 6 (0.0%) | 10 (0.1%) | 10 (0.1%) | 26 (0.1%) |
| Candida sp. (Not albicans) | 1 (0.0%) | 6 (0.1%) | 0 (0%) | 7 (0.0%) |
| Candida tropicalis | 15 (0.1%) | 27 (0.2%) | 21 (0.1%) | 63 (0.1%) |
| Chryseobacterium gleum | 1 (0.0%) | 0 (0%) | 1 (0.0%) | 2 (0.0%) |
| Chryseobacterium indologenes | 2 (0.0%) | 1 (0.0%) | 0 (0%) | 3 (0.0%) |
| Citrobacter braakii | 1 (0.0%) | 0 (0%) | 0 (0%) | 1 (0.0%) |
| Citrobacter freundii | 36 (0.1%) | 17 (0.1%) | 28 (0.2%) | 81 (0.2%) |
| Citrobacter koseri | 105 (0.4%) | 91 (0.8%) | 74 (0.5%) | 270 (0.5%) |
| Citrobacter sedlakii | 1 (0.0%) | 0 (0%) | 0 (0%) | 1 (0.0%) |
| Citrobacter sp. | 1 (0.0%) | 0 (0%) | 1 (0.0%) | 2 (0.0%) |
| Coag Neg Staphylococcus | 9 (0.0%) | 8 (0.1%) | 4 (0.0%) | 21 (0.0%) |
| Coliform bacillus | 76 (0.3%) | 31 (0.3%) | 26 (0.2%) | 133 (0.3%) |
| Coryne pseudodiphtheriticum | 1 (0.0%) | 4 (0.0%) | 3 (0.0%) | 8 (0.0%) |
| Corynebacterium amycolatum | 1 (0.0%) | 0 (0%) | 0 (0%) | 1 (0.0%) |
| Corynebacterium diphtheriae | 1 (0.0%) | 0 (0%) | 0 (0%) | 1 (0.0%) |
| Corynebacterium propinquum | 7 (0.0%) | 3 (0.0%) | 6 (0.0%) | 16 (0.0%) |
| Corynebacterium sp. | 2 (0.0%) | 0 (0%) | 2 (0.0%) | 4 (0.0%) |
| Corynebacterium striatum | 236 (0.9%) | 169 (1.5%) | 117 (0.8%) | 522 (1.0%) |
| Cryptococcus sp. | 1 (0.0%) | 0 (0%) | 0 (0%) | 1 (0.0%) |
| Cupriavidus species | 1 (0.0%) | 0 (0%) | 0 (0%) | 1 (0.0%) |
| Delftia acidovorans | 3 (0.0%) | 1 (0.0%) | 1 (0.0%) | 5 (0.0%) |
| Elizabethkingia miricola | 1 (0.0%) | 2 (0.0%) | 0 (0%) | 3 (0.0%) |
| Enterobacter cloacae | 212 (0.8%) | 118 (1.0%) | 120 (0.9%) | 450 (0.9%) |
| Enterobacter sp. | 2 (0.0%) | 1 (0.0%) | 2 (0.0%) | 5 (0.0%) |
| Enterococcus faecalis | 9 (0.0%) | 4 (0.0%) | 2 (0.0%) | 15 (0.0%) |
| Enterococcus faecium | 1 (0.0%) | 2 (0.0%) | 2 (0.0%) | 5 (0.0%) |
| Enterococcus sp. | 30 (0.1%) | 16 (0.1%) | 8 (0.1%) | 54 (0.1%) |
| Escherichia coli | 857 (3.4%) | 576 (4.9%) | 463 (3.3%) | 1896 (3.7%) |
| Escherichia vulneris | 1 (0.0%) | 0 (0%) | 0 (0%) | 1 (0.0%) |
| Ewingella americana | 1 (0.0%) | 1 (0.0%) | 0 (0%) | 2 (0.0%) |
| Exiguobacterium aurantiacum | 1 (0.0%) | 0 (0%) | 0 (0%) | 1 (0.0%) |
| Group A streptococcus | 106 (0.4%) | 32 (0.3%) | 102 (0.7%) | 240 (0.5%) |
| Group B streptococcus | 73 (0.3%) | 30 (0.3%) | 23 (0.2%) | 126 (0.2%) |
| Group C streptococcus | 54 (0.2%) | 19 (0.2%) | 30 (0.2%) | 103 (0.2%) |
| Group G streptococcus | 76 (0.3%) | 40 (0.3%) | 45 (0.3%) | 161 (0.3%) |
| Haemophilus haemolyticus | 1 (0.0%) | 0 (0%) | 0 (0%) | 1 (0.0%) |
| Haemophilus influenzae | 10855 (43.3%) | 4137 (35.5%) | 6185 (43.9%) | 21177 (41.7%) |
| Haemophilus parainfluenzae | 38 (0.2%) | 5 (0.0%) | 2 (0.0%) | 45 (0.1%) |
| Haemophilus sp | 2 (0.0%) | 1 (0.0%) | 2 (0.0%) | 5 (0.0%) |
| Hafnia alvei | 5 (0.0%) | 10 (0.1%) | 23 (0.2%) | 38 (0.1%) |
| Klebsiella aerogenes | 76 (0.3%) | 63 (0.5%) | 56 (0.4%) | 195 (0.4%) |
| Klebsiella oxytoca | 158 (0.6%) | 79 (0.7%) | 95 (0.7%) | 332 (0.7%) |
| Klebsiella pneumoniae | 476 (1.9%) | 261 (2.2%) | 321 (2.3%) | 1058 (2.1%) |
| Klebsiella sp. | 3 (0.0%) | 2 (0.0%) | 0 (0%) | 5 (0.0%) |
| Klebsiella species | 2 (0.0%) | 0 (0%) | 0 (0%) | 2 (0.0%) |
| Kluyvera intermedia | 1 (0.0%) | 1 (0.0%) | 0 (0%) | 2 (0.0%) |
| Legionella pneumophila | 5 (0.0%) | 0 (0%) | 2 (0.0%) | 7 (0.0%) |
| Lelliottia amnigena | 2 (0.0%) | 0 (0%) | 0 (0%) | 2 (0.0%) |
| Leuconostoc mesenteroides | 1 (0.0%) | 0 (0%) | 0 (0%) | 1 (0.0%) |
| Lysinibacillus fusiformis | 1 (0.0%) | 0 (0%) | 0 (0%) | 1 (0.0%) |
| Meyerozyma guilliermondii | 1 (0.0%) | 2 (0.0%) | 0 (0%) | 3 (0.0%) |
| Microvirgula aerodentrificans | 1 (0.0%) | 0 (0%) | 0 (0%) | 1 (0.0%) |
| Mixed coag.neg. staphylococci | 1 (0.0%) | 0 (0%) | 2 (0.0%) | 3 (0.0%) |
| Mixed Coliforms | 131 (0.5%) | 40 (0.3%) | 57 (0.4%) | 228 (0.4%) |
| Moraxella catarrhalis | 2503 (10.0%) | 790 (6.8%) | 1358 (9.6%) | 4651 (9.2%) |
| Moraxella osloensis | 1 (0.0%) | 0 (0%) | 2 (0.0%) | 3 (0.0%) |
| Moraxella sp | 1 (0.0%) | 0 (0%) | 0 (0%) | 1 (0.0%) |
| Morganella morganii | 32 (0.1%) | 26 (0.2%) | 31 (0.2%) | 89 (0.2%) |
| MRSA | 69 (0.3%) | 44 (0.4%) | 56 (0.4%) | 169 (0.3%) |
| Mucoid Pseudomonas | 228 (0.9%) | 78 (0.7%) | 39 (0.3%) | 345 (0.7%) |
| Neisseria cinerea | 1 (0.0%) | 0 (0%) | 0 (0%) | 1 (0.0%) |
| Neisseria meningitidis | 146 (0.6%) | 33 (0.3%) | 60 (0.4%) | 239 (0.5%) |
| Nocardia cyriacigeorgica | 2 (0.0%) | 0 (0%) | 0 (0%) | 2 (0.0%) |
| Non-mucoid Pseudomonas | 1 (0.0%) | 0 (0%) | 0 (0%) | 1 (0.0%) |
| Pantoea agglomerans | 2 (0.0%) | 0 (0%) | 1 (0.0%) | 3 (0.0%) |
| Pantoea septica | 1 (0.0%) | 1 (0.0%) | 0 (0%) | 2 (0.0%) |
| Pasteurella canis | 18 (0.1%) | 6 (0.1%) | 10 (0.1%) | 34 (0.1%) |
| Pasteurella dagmatis | 3 (0.0%) | 0 (0%) | 0 (0%) | 3 (0.0%) |
| Pasteurella multocida | 23 (0.1%) | 14 (0.1%) | 6 (0.0%) | 43 (0.1%) |
| Pasteurella stomatis | 2 (0.0%) | 1 (0.0%) | 0 (0%) | 3 (0.0%) |
| Penicillium sp. | 2 (0.0%) | 19 (0.2%) | 2 (0.0%) | 23 (0.0%) |
| Pluralibacter gergoviae | 2 (0.0%) | 6 (0.1%) | 3 (0.0%) | 11 (0.0%) |
| Proteus hauseri | 2 (0.0%) | 0 (0%) | 0 (0%) | 2 (0.0%) |
| Proteus mirabilis | 225 (0.9%) | 143 (1.2%) | 173 (1.2%) | 541 (1.1%) |
| Proteus sp. | 10 (0.0%) | 3 (0.0%) | 2 (0.0%) | 15 (0.0%) |
| Proteus vulgaris | 2 (0.0%) | 5 (0.0%) | 1 (0.0%) | 8 (0.0%) |
| Providencia rettgeri | 3 (0.0%) | 2 (0.0%) | 7 (0.0%) | 12 (0.0%) |
| Providencia stuartii | 1 (0.0%) | 1 (0.0%) | 0 (0%) | 2 (0.0%) |
| Pseudomonas aeruginosa | 2221 (8.9%) | 1566 (13.4%) | 1221 (8.7%) | 5008 (9.9%) |
| Pseudomonas fluorescens | 5 (0.0%) | 2 (0.0%) | 0 (0%) | 7 (0.0%) |
| Pseudomonas fragi | 1 (0.0%) | 0 (0%) | 0 (0%) | 1 (0.0%) |
| Pseudomonas koreensis | 3 (0.0%) | 2 (0.0%) | 2 (0.0%) | 7 (0.0%) |
| Pseudomonas nitroreducens | 1 (0.0%) | 0 (0%) | 0 (0%) | 1 (0.0%) |
| Pseudomonas putida | 15 (0.1%) | 7 (0.1%) | 17 (0.1%) | 39 (0.1%) |
| Pseudomonas rhodesiae | 1 (0.0%) | 0 (0%) | 0 (0%) | 1 (0.0%) |
| Pseudomonas sp. | 50 (0.2%) | 15 (0.1%) | 11 (0.1%) | 76 (0.1%) |
| Pseudomonas stutzeri | 6 (0.0%) | 2 (0.0%) | 3 (0.0%) | 11 (0.0%) |
| Rahnella aquatilis | 3 (0.0%) | 0 (0%) | 0 (0%) | 3 (0.0%) |
| Raoultella ornithinolytica | 26 (0.1%) | 18 (0.2%) | 20 (0.1%) | 64 (0.1%) |
| Raoultella planticola | 3 (0.0%) | 2 (0.0%) | 2 (0.0%) | 7 (0.0%) |
| Raoultella species | 6 (0.0%) | 3 (0.0%) | 10 (0.1%) | 19 (0.0%) |
| Rothia mucilaginosa | 13 (0.1%) | 1 (0.0%) | 1 (0.0%) | 15 (0.0%) |
| Saccharomyces cerevisiae | 2 (0.0%) | 7 (0.1%) | 4 (0.0%) | 13 (0.0%) |
| Saccharomyces sp. | 1 (0.0%) | 0 (0%) | 1 (0.0%) | 2 (0.0%) |
| Serratia liquefaciens | 36 (0.1%) | 10 (0.1%) | 26 (0.2%) | 72 (0.1%) |
| Serratia marcescens | 373 (1.5%) | 230 (2.0%) | 264 (1.9%) | 867 (1.7%) |
| Serratia plymuthica | 1 (0.0%) | 0 (0%) | 0 (0%) | 1 (0.0%) |
| Serratia proteamaculans | 1 (0.0%) | 1 (0.0%) | 0 (0%) | 2 (0.0%) |
| Serratia rubidaea | 2 (0.0%) | 1 (0.0%) | 0 (0%) | 3 (0.0%) |
| Serratia sp. | 5 (0.0%) | 5 (0.0%) | 9 (0.1%) | 19 (0.0%) |
| Serratia species | 3 (0.0%) | 2 (0.0%) | 28 (0.2%) | 33 (0.1%) |
| Staphylococcus aureus | 2028 (8.1%) | 1269 (10.9%) | 1161 (8.2%) | 4458 (8.8%) |
| Stenotrophomonas maltophilia | 318 (1.3%) | 145 (1.2%) | 126 (0.9%) | 589 (1.2%) |
| Streptococcus agalactiae | 2 (0.0%) | 0 (0%) | 0 (0%) | 2 (0.0%) |
| Streptococcus dysgalactiae | 2 (0.0%) | 0 (0%) | 0 (0%) | 2 (0.0%) |
| Streptococcus milleri | 16 (0.1%) | 11 (0.1%) | 5 (0.0%) | 32 (0.1%) |
| Streptococcus parasanguis | 1 (0.0%) | 0 (0%) | 0 (0%) | 1 (0.0%) |
| Streptococcus pneumoniae | 2434 (9.7%) | 729 (6.3%) | 1057 (7.5%) | 4220 (8.3%) |
| Streptococcus pseudopneumoniae | 4 (0.0%) | 1 (0.0%) | 0 (0%) | 5 (0.0%) |
| Viridans streptococci | 1 (0.0%) | 0 (0%) | 0 (0%) | 1 (0.0%) |
| Yeasts | 2 (0.0%) | 0 (0%) | 0 (0%) | 2 (0.0%) |
| Acinetobacter beijerinckii | 0 (0%) | 2 (0.0%) | 0 (0%) | 2 (0.0%) |
| Acinetobacter bereziniae | 0 (0%) | 1 (0.0%) | 0 (0%) | 1 (0.0%) |
| Acinetobacter dispersus | 0 (0%) | 1 (0.0%) | 1 (0.0%) | 2 (0.0%) |
| Actinobacillus suis | 0 (0%) | 1 (0.0%) | 1 (0.0%) | 2 (0.0%) |
| Aeromonas caviae | 0 (0%) | 1 (0.0%) | 0 (0%) | 1 (0.0%) |
| Aspergillus flavus | 0 (0%) | 5 (0.0%) | 3 (0.0%) | 8 (0.0%) |
| Bacillus simplex | 0 (0%) | 1 (0.0%) | 0 (0%) | 1 (0.0%) |
| Bacillus subtilis | 0 (0%) | 1 (0.0%) | 0 (0%) | 1 (0.0%) |
| Burkholderia species | 0 (0%) | 1 (0.0%) | 0 (0%) | 1 (0.0%) |
| Citrobacter gillenii | 0 (0%) | 1 (0.0%) | 0 (0%) | 1 (0.0%) |
| Environmental fungus | 0 (0%) | 4 (0.0%) | 0 (0%) | 4 (0.0%) |
| Escherichia hermannii | 0 (0%) | 1 (0.0%) | 0 (0%) | 1 (0.0%) |
| Fusarium sp. | 0 (0%) | 1 (0.0%) | 0 (0%) | 1 (0.0%) |
| Kocuria sp | 0 (0%) | 1 (0.0%) | 0 (0%) | 1 (0.0%) |
| Leclercia adecarboxylata | 0 (0%) | 1 (0.0%) | 1 (0.0%) | 2 (0.0%) |
| Micrococcus sp. | 0 (0%) | 1 (0.0%) | 0 (0%) | 1 (0.0%) |
| Mixed Candida sp. | 0 (0%) | 1 (0.0%) | 0 (0%) | 1 (0.0%) |
| Neisseria subflava | 0 (0%) | 1 (0.0%) | 0 (0%) | 1 (0.0%) |
| Pandoraea apista | 0 (0%) | 5 (0.0%) | 3 (0.0%) | 8 (0.0%) |
| Pandoraea sp. | 0 (0%) | 1 (0.0%) | 1 (0.0%) | 2 (0.0%) |
| Pasteurella sp | 0 (0%) | 1 (0.0%) | 0 (0%) | 1 (0.0%) |
| Pichia sp. | 0 (0%) | 1 (0.0%) | 0 (0%) | 1 (0.0%) |
| Ralstonia insidiosa | 0 (0%) | 2 (0.0%) | 0 (0%) | 2 (0.0%) |
| Scedosporium apiospermum | 0 (0%) | 1 (0.0%) | 5 (0.0%) | 6 (0.0%) |
| Staph. epidermidis | 0 (0%) | 1 (0.0%) | 0 (0%) | 1 (0.0%) |
| Staph. lugdunensis | 0 (0%) | 1 (0.0%) | 0 (0%) | 1 (0.0%) |
| Streptococcus gallolyticus | 0 (0%) | 1 (0.0%) | 0 (0%) | 1 (0.0%) |
| Streptococcus oralis | 0 (0%) | 1 (0.0%) | 0 (0%) | 1 (0.0%) |
| Acinetobacter parvus | 0 (0%) | 0 (0%) | 1 (0.0%) | 1 (0.0%) |
| Actinotignum species | 0 (0%) | 0 (0%) | 1 (0.0%) | 1 (0.0%) |
| Aspergillus candidus | 0 (0%) | 0 (0%) | 1 (0.0%) | 1 (0.0%) |
| Aspergillus terreus | 0 (0%) | 0 (0%) | 1 (0.0%) | 1 (0.0%) |
| Bordetella parapertussis | 0 (0%) | 0 (0%) | 1 (0.0%) | 1 (0.0%) |
| Burkholderia multivorans | 0 (0%) | 0 (0%) | 1 (0.0%) | 1 (0.0%) |
| Burkholderia pseudomallei | 0 (0%) | 0 (0%) | 1 (0.0%) | 1 (0.0%) |
| Candida metapsilosis | 0 (0%) | 0 (0%) | 2 (0.0%) | 2 (0.0%) |
| Cedecea neteri | 0 (0%) | 0 (0%) | 1 (0.0%) | 1 (0.0%) |
| Clavispora lusitaniae | 0 (0%) | 0 (0%) | 6 (0.0%) | 6 (0.0%) |
| Corynebacterium sp | 0 (0%) | 0 (0%) | 2 (0.0%) | 2 (0.0%) |
| Exophiala dermatitidis | 0 (0%) | 0 (0%) | 2 (0.0%) | 2 (0.0%) |
| Gemella haemolysans | 0 (0%) | 0 (0%) | 1 (0.0%) | 1 (0.0%) |
| Haemophilus sputorum | 0 (0%) | 0 (0%) | 1 (0.0%) | 1 (0.0%) |
| Hanseniaspora opuntiae | 0 (0%) | 0 (0%) | 1 (0.0%) | 1 (0.0%) |
| Kluyvera cryocrescens | 0 (0%) | 0 (0%) | 1 (0.0%) | 1 (0.0%) |
| Kluyveromyces marxianus | 0 (0%) | 0 (0%) | 4 (0.0%) | 4 (0.0%) |
| Pichia kudriavazevii/C krusei | 0 (0%) | 0 (0%) | 2 (0.0%) | 2 (0.0%) |
| Pichia kudriavzevii | 0 (0%) | 0 (0%) | 5 (0.0%) | 5 (0.0%) |
| Providencia vermicola | 0 (0%) | 0 (0%) | 1 (0.0%) | 1 (0.0%) |
| Pseud. aeruginosa (mucoid) | 0 (0%) | 0 (0%) | 5 (0.0%) | 5 (0.0%) |
| Pseudomonas citronellolis | 0 (0%) | 0 (0%) | 1 (0.0%) | 1 (0.0%) |
| Pseudomonas jinjuensis | 0 (0%) | 0 (0%) | 1 (0.0%) | 1 (0.0%) |
| Unidentified | 135 (0.5%) | 4 (0.0%) | 2 (0.0%) | 141 (0.3%) |
| Wickerhamomyces anomalus | 0 (0%) | 0 (0%) | 1 (0.0%) | 1 (0.0%) |
| Yersinia sp. | 0 (0%) | 0 (0%) | 1 (0.0%) | 1 (0.0%) |

Table S4. Streptococcus sp.

| **Organisms** | **Before COVID (N=2769)** | **During COVID (N=862)** | **After COVID (N=1262)** | **Overall (N=4893)** |
| --- | --- | --- | --- | --- |
| Group A streptococcus | 106 (3.8%) | 32 (3.7%) | 102 (8.1%) | 240 (4.9%) |
| Group B streptococcus | 73 (2.6%) | 30 (3.5%) | 23 (1.8%) | 126 (2.6%) |
| Group C streptococcus | 54 (2.0%) | 19 (2.2%) | 30 (2.4%) | 103 (2.1%) |
| Group G streptococcus | 76 (2.7%) | 40 (4.6%) | 45 (3.6%) | 161 (3.3%) |
| Streptococcus agalactiae | 2 (0.1%) | 0 (0%) | 0 (0%) | 2 (0.0%) |
| Streptococcus dysgalactiae | 2 (0.1%) | 0 (0%) | 0 (0%) | 2 (0.0%) |
| Streptococcus milleri | 16 (0.6%) | 11 (1.3%) | 5 (0.4%) | 32 (0.7%) |
| Streptococcus parasanguis | 1 (0.0%) | 0 (0%) | 0 (0%) | 1 (0.0%) |
| Streptococcus pneumoniae | 2434 (87.9%) | 729 (84.6%) | 1057 (83.8%) | 4220 (86.2%) |
| Streptococcus pseudopneumoniae | 4 (0.1%) | 1 (0.1%) | 0 (0%) | 5 (0.1%) |
| Viridans streptococci | 1 (0.0%) | 0 (0%) | 0 (0%) | 1 (0.0%) |

Table S5. Percent difference in Streptococcus sp. cultured during each period of the study

| Cultured organisms | **During COVID**  **vs**  **Before COVID** | | **After COVID**  **vs**  **During COVID** | | **After COVID**  **vs**  **Before COVID** | |
| --- | --- | --- | --- | --- | --- | --- |
|  | % difference | *P value* | % difference | *P value* | % difference | *P value* |
| Streptococcus pneumoniae | -40.4 | <0.001 | 7.7 | <0.001 | -32.7 | <0.001 |
| Group A streptococcus | -30.9 | <0.001 | 29.2 | <0.001 | -1.7 | 0.7823 |
| Group B streptococcus | -34.1 | <0.001 | -5.5 | 0.3537 | 39.6 | <0.001 |
| Group C streptococcus | -34.0 | <0.001 | 10.7 | 0.1018 | -23.3 | 0.0011 |
| Group G streptococcus | -22.4 | <0.001 | 3.2 | 0.6131 | -19.2 | 0.0010 |

(a)

(b)

Figure S3. Proportion of isolates for each study period in (a) community and acute settings, (b) inpatient settings.
